# Association of Pain, Insomnia, and Depressive Symptom Clusters with Cognitive Decline

**DOI:** 10.1093/geroni/igaf122.3543

**Published:** 2025-12-31

**Authors:** Matthew Phan, Utpol Das, Tyler Bell, Mukaila Raji, Sadaf Milani

**Affiliations:** University of Texas Medical Branch, Galveston, Texas, United States; University of Texas Medical Branch, Galveston, Texas, United States; University of California, San Diego, San Diego, California, United States; University of Texas Medical Branch, Galveston, Texas, United States; University of Texas Medical Branch, Galveston, Texas, United States

## Abstract

Pain-Insomnia-and-Depression syndrome (PIDS) refers to the triad of chronic pain, sleep disturbances, and depressive symptoms disproportionately affecting older adults. Limited data exist on the rates and patterns of these symptom clusters and their association with cognitive function. Our objectives were to characterize symptom clusters of pain, insomnia, and depression and to examine their association with cognitive decline among middle-aged and older Americans. We used data from the Health and Retirement Study (2010-2018), a nationally representative longitudinal study of Americans over age 50 (n = 18,599). Latent class analysis identified clusters based on pain prevalence, insomnia, and depressive symptoms. Mixed-effects linear regression models examined the associations between these clusters and cognitive decline. Five clusters were identified: 1) minimal symptoms (56.90%), 2) moderate pain, low insomnia, low depressive symptoms (7.3%), 3) no pain, high insomnia, moderate depressive symptoms (6.4%), 4) high-impact pain, moderate insomnia, moderate depressive symptoms (11.8%), and 5) high-impact pain, high insomnia, high depressive symptoms (7.5%). Compared to those with minimal symptoms, individuals in the “no pain, high insomnia, and moderate depression” and “high-impact pain, moderate insomnia, and moderate depression” clusters experienced the fastest rates of cognitive decline (β= -0.07, 95% CI: -0.13, -0.01; β= -0.07, 95% CI: -0.11, -0.02, respectively). These results suggest a complex interplay among pain, insomnia, and depression, particularly in relation to the development of cognitive decline in older age. Screening for co-occurring symptoms may inform therapeutic clinical decision-making aimed at mitigating cognitive decline.

